# Data-intensive immune network modelling for One Health

**DOI:** 10.1093/bib/bbag420

**Published:** 2026-08-06

**Authors:** Richard Hillis, Nadya B A Johari, Masoud Shirali, Ian M Overton

**Affiliations:** Johnston Cancer Research Centre, Queen's University Belfast, Belfast, UK; Agri-Food and Biosciences Institute, Hillsborough, UK; Johnston Cancer Research Centre, Queen's University Belfast, Belfast, UK; Agri-Food and Biosciences Institute, Hillsborough, UK; School of Biological Sciences, Queen's University Belfast, Belfast, UK; Johnston Cancer Research Centre, Queen's University Belfast, Belfast, UK

**Keywords:** immune system, gene expression, transcriptomics, GWAS, single-cell, zoonoses, cancer, network biology, machine learning

## Abstract

The immune system plays a critical role in morbidity and mortality. For example, infectious disease, cancer, and autoimmune disorders impose substantial health burdens upon both humans and animals. Immune systems share fundamental organisation, and the transmission of pathogens across species boundaries means that immune health in one population shapes risk in others. The One Health approach recognises this interdependence as the basis for understanding human, animal, and environmental health together. Immune systems are formed from multiscale networks of biomolecular interactions spanning specialised cell types, dynamic states, and differentiation trajectories. Data-intensive computational methods are required to model these processes accurately in specific biological contexts. Accordingly, bioinformatics is essential for understanding how the immune system functions under various conditions that arise from infections, in chronic diseases, and through environmental exposures. This article reviews cutting-edge techniques for studying immune function in human and animal health; with emphasis upon genetics, transcriptomics, single-cell approaches, network biology, and machine learning. We consider bioinformatics applications that inform our understanding of immune function to improve health and food systems. Examples are discussed from the rapidly developing cross-disciplinary landscape of computational and physical techniques. We illustrate data-intensive approaches in understanding context-specific immune biology, applied to illuminate the relationship between genetic variation and disease phenotypes.

## Introduction

The immune system provides protection against infectious disease, cancer, and autoimmune disorders [1–3], functioning through complex interactions across specialised cell types [1–7]. Immune cell types and their regulatory relationships are broadly conserved across mammals, including economically important farm animals such as dairy cattle [2–4, 8]. There are also differences in immune network architectures and pathogen responses between species, reflecting distinct ecological and evolutionary pressures [2–6, 8–13]. Understanding conserved and divergent features is critical for adapting knowledge developed in animals to human medicine and vice versa. Key functions of the immune system are to identify and eliminate foreign objects or pathogens, as well as infected or cancerous cells, carried out through both antibody and cell-mediated responses in the innate and adaptive branches [1–4, 7, 8, 14–19]. The innate immune system provides the initial defence through dendritic cells, natural killer cells, macrophages, neutrophils, basophils, eosinophils, and mast cells; while the adaptive immune system, directed by B and T lymphocytes, targets pathogens that escape innate responses [1–6, 8].

Network biology provides a powerful framework to understand complex biological systems, drawing on graph theory, systems biology, computer science, and mathematics [5, 20–25]. Biological networks represent nodes such as genes, proteins, or metabolites connected by edges signifying physical or functional interactions. These networks typically feature highly connected hub nodes and community modules corresponding to biological pathways or protein complexes [3, 5, 19–25]. Transcriptome data is widely used for network construction, for example in co-expression and gene regulatory networks, and network analysis can inform discovery of disease-associated genes, biomarkers, and drug targets [19, 20, 23–27].

A key limitation of data from bulk tissue is the lack of single-cell resolution, which is critical for understanding context-specific immune function. Bulk samples provide an averaged expression profile that obscures contributions of specific cell types and states, leading to inaccurate network inference [3–5, 7, 14, 15, 17, 24, 28–30]. This is particularly consequential in cancer, where tumours are highly heterogeneous, the tumour microenvironment shapes treatment response, and the immune system plays dual roles in both suppressing and promoting tumour progression [14, 15, 17–19, 31–35]. Single-cell technologies address this limitation, enabling characterisation of cellular heterogeneity and rare cell types, and mapping of immune developmental trajectories [6, 7, 14, 15, 24, 28, 29, 36–39].

**Figure 1:**
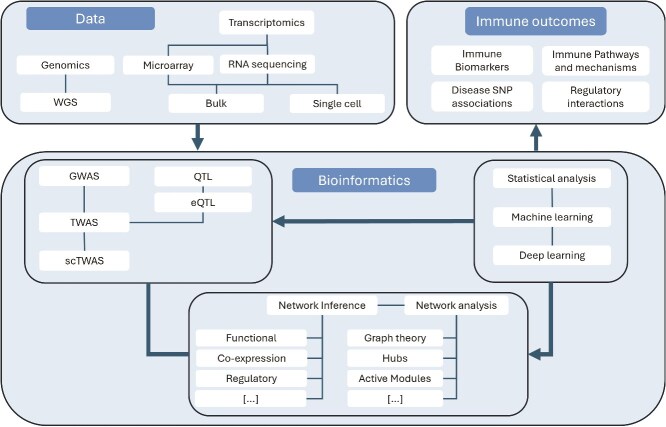
Modelling the immune system for one health with data-intensive techniques. Applications with genomic and transcriptomic data are considered, where bioinformatics techniques can inform immune outcomes relevant to human and veterinary medicine. Bioinformatics analyses, including genome-wide association studies (GWAS) and related approaches, statistical modelling, network analysis, and machine learning are powerful tools for driving forwards biomedical discoveries. These approaches are interrelated; for example, machine learning may enable network inference, while networks may enhance GWAS for example in NetWAS. Applications of these methods include understanding the relationship between genotype and phenotype, revealing multiscale immune mechanisms, guiding biomarker development and therapeutic interventions towards precision health.

This review explores immune function through ‘omics technologies and network biology approaches with examples from human and animal health. We consider three interconnected developments that, taken together, may shape data-intensive immune modelling for One Health [3, 6, 19, 21, 38, 40]. The first is the shift from bulk-tissue to cell-type-resolved network inference, motivated by the recognition that immune function is determined by cell-state-specific regulatory programmes that bulk averaging tends to obscure [5, 7, 24, 41]. The second is the emergence of multi-scale executable models, including agent-based models (ABMs), virtual cells, and digital twins, as a bridge between molecular networks and population-level epidemiological simulations [42–46]. The third is the growing use of foundation models and transfer learning to propagate biological knowledge to data-poor contexts, including non-model species relevant to veterinary medicine and zoonotic surveillance [47–49]. The remainder of this review considers progress along each direction, alongside persistent obstacles in data sparsity, cross-species extrapolation, and gold-standard alignment (Figure 1).

## Immune function in human and animal health

The immune mechanisms introduced above operate in the context of a shared biological landscape spanning humans, animals, and their environments. Human and animal health are governed by interconnected multiscale systems where immune function plays a key role [1–5, 21]. Domesticated animals such as dairy cattle are important for the agricultural sector and are also potential vectors for zoonotic and antibiotic-resistant diseases [2, 50–53]. The immune system is largely conserved across mammals in terms of cell types and regulatory pathways [1–4, 6]; however, differences in immune network architecture between species influence susceptibility to disease and treatment outcomes [9, 10, 12, 13], underscoring the need to account for species-specific dynamics when addressing zoonotic infections or improving herd health [50–54].

Zoonoses mainly affect those in close proximity to animals, but exposure can also occur through contaminated animal products [50–53]. Well-known examples include bovine tuberculosis and foot-and-mouth disease, while Transboundary Animal Diseases can spread rapidly between wildlife and domesticated species, crossing national borders and persisting in wild reservoir populations [53, 55–57]. The spread of antibiotic-resistant disease is a particularly important concern; isolates resistant to treatment occur across multiple species, farm locations, and in humans, including MRSA, MDR Shiga toxin-producing Escherichia coli, and streptomycin-resistant M. bovis [50, 52, 53, 58–60]. Beyond direct zoonotic risks, diseases in cattle decrease milk production and quality, hindering nutrition and damaging farmer livelihoods. These relationships are increasingly consequential considering the demand from a rising global population and the potential impacts of climate change, which may expand the geographical range of pathogens and negatively affect immune performance through heat stress and other environmental stressors [2, 50, 53, 61–68].

The immune system is deeply involved in cancer through immunoediting, which occurs in three main stages [17, 32, 69, 70]. In the elimination stage, coordinated innate and adaptive responses target tumour cells, involving natural killer cells, macrophages, dendritic cells and lymphocytes [17, 32, 71]. Failure to achieve complete elimination leads to an equilibrium stage, during which remaining tumour cells acquire the ability to survive or evade immune interactions [32, 71]. In the escape stage, tumours have developed the “three Cs”: camouflage, where tumours avoid immune detection; coercion, where they suppress or manipulate immune cells; and cytoprotection, which prevents destruction by immune attacks, a hallmark of cancer [32, 69]. Accordingly, cancer progression involves context-specific “rewiring” of cell functions. For instance, granulocytes contribute to tumour destruction responses by generating reactive oxygen species, secretion of inflammatory cytokines, and release of toxic moieties; however, granulocytes can also be exploited by tumour cells, acting to facilitate cancer development, progression, and metastasis as well as inactivating the immune response [31]. The local environment influences cancer risk, including presence of carcinogenic substances, dietary factors and infection by certain viral pathogens. Furthermore, weakening immune health as a direct result of cancer and treatments such as radio- and chemotherapy, can make patients more susceptible to infection [72–75].

There are clear connections between human immune health, the immune systems and diseases of other species, and our shared environments. These interdependencies underlie the One Health approach, which recognises the relationships between the health of people and animals, as well as the influence of the environment [50–53, 72, 76]. Hence, appropriate analysis methods are required to understand these intertwined systems across different scales. Network-based approaches may be particularly well suited for this task. Analysis at single-cell resolution is important for elucidating the context-specific functioning of the immune system, as well as in advancing our understanding of genetic factors affecting immune health and disease response [3, 5–7, 14, 15, 36]. Techniques used to study immune cell networks continue to develop in tandem with emerging multiomics technologies and associated statistical and computational approaches [3, 5–7, 14, 19, 24, 36, 37, 40, 41, 77]. The ‘omics data generated by these technologies, from genome sequences to single-cell transcriptomes, form the empirical substrate from which immune network models are constructed; the following sections consider these data types and the analytical approaches that transform them into biological insight.

### Genetics and functional genomics in immune research

Genetic data has widespread applications, including assessing individual disease risk and identifying and selecting for improved productive traits in animal breeding [61, 78–81]. Genome wide association studies (GWAS) involve genotyping of test and control cohorts, commonly using SNP arrays or sequence-imputed genotypes, to identify SNPs associated with a phenotype; regions of high linkage disequilibrium with associated SNPs may reveal candidate genes controlling the observed phenotypic variation [57, 82, 83]. This approach has been applied across multiple species, including health and production traits in domesticated animals. However, analysis restricted to genome data alone has limitations, for example arising from the lack of information about context-specific gene expression and the challenges with assigning biological function to non-coding SNPs [57, 82–85]. Transcriptome sequencing including RNA-seq and miRNA assays, alongside methods for measuring chromatin state such as ATAC-seq are helpful for genome interpretation [84–93].

**Figure 2:**
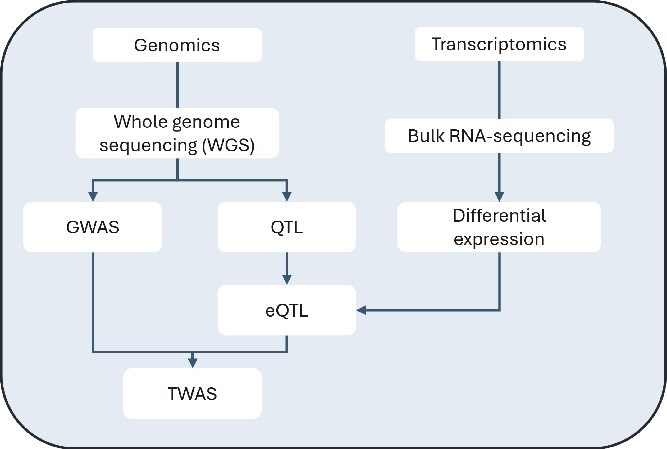
Analysis and integration of bulk genomics and transcriptomics data. Multiple methods have been developed for integration of genomics and transcriptomics data such as eQTL and TWAS analysis. The flowchart summarises common analysis approaches for both data types.

An important consideration in both GWAS and standard transcriptomics is that individual genes or SNPs often only have small effect sizes, with complex traits typically driven by the action of many variants [94–96]. The multifactorial nature of genotype-phenotype relationships is underlined by the power of polygenic risk scores [97–99]. Integrative approaches combining differential expression with GWAS, expression quantitative trait loci (eQTL) methods, and transcriptome-wide association studies (TWAS) address these limitations by linking SNPs in non-coding regions to changes in gene expression associated with a trait and have been applied in human diseases such as silicosis and lymphoma, as well as in cattle for milk protein content and ketosis [85, 92, 100–105]. Network analysis can integrate these signals at the level of pathways and protein complexes; an early example is NetWAS [106] (Figure 2). TWAS advances upon the eQTL approach by using regulatory information from eQTLs to build models of gene expression, estimating transcript levels from genotype and correlating with phenotype across large GWAS datasets [85, 104, 105, 107]. Accordingly, TWAS allows tissue-specific analysis, mitigates potentially limited availability of transcriptome data, and enhances statistical power by reducing multiple hypothesis testing relative to GWAS [85, 104, 105]. Multigroup causal TWAS (M-cTWAS) extends this framework to integrate QTL datasets across several ‘omics modalities and tissues, with demonstrated advantages in heritability explanation, false discovery control, and causal gene identification [108]. Single-cell TWAS approaches such as CONTENT [109] and EXPRESSO [110] enable tissue- and cell-type-specific analysis, enhancing statistical power by reducing multiple hypothesis testing, with a key advantage being the identification of cell-type-specific effects and signals that may be inaccessible in analysis of bulk RNA [85, 106, 111, 112].

While these techniques are powerful, they do not fully address immune cell heterogeneity. Cellular heterogeneity is lost or “blende-rised” in bulk RNA sequencing, which averages expression values across cell types, activation states, and differentiation trajectories, leading to information loss [7, 14, 24, 29, 113–116]. Technologies that assess gene expression at single-cell resolution therefore have clear advantages for the study of immune mechanisms. Understanding how genetic variation shapes gene expression in specific cell types motivates the single-cell approaches considered in the following section.

### Single-cell transcriptome profiling

Single-cell transcriptomics provides a foundation for much of what follows in this review; network models, multi-scale simulations, and cross-species transfer methods are considerably more informative when cell identities and states may be resolved [3, 6, 7, 15, 24, 36, 41]. Since the first application to mammalian cells in 2009, scRNA-seq has been deployed for characterisation of cellular heterogeneity, identification of rare cell types, and mapping of immune developmental trajectories [7, 14, 24, 30, 36, 38, 114–116]. Methods are now available for single-cell proteomics, metabolomics, and epigenomics; multi-modal and spatially resolved approaches have also been developed [37, 40]. The workflow begins with capture and separation of individual cells, barcoding of mRNA from each cell, conversion to cDNA by reverse transcription, amplification, and library preparation for sequencing [6, 29, 37, 40, 117]. CellRanger and Seurat are popular tools for processing scRNA-seq data [38, 117]; Monocle, Scanpy, and GF-ICF are also widely used [29, 118–120]. Key steps are filtering “low-quality” genes and cells, normalisation, and unsupervised cell clustering [29, 37, 38]. Ensuring the integrity of downstream analysis requires application of proper quality control and normalisation methods to detect and mitigate biases, which may be more prevalent in single-cell than bulk data [29, 37, 38]. scRNA-seq allows identification of cell type distributions and discovery of new candidate patient subgroups that might correlate with disease progression and prognosis [3, 7, 14, 15, 24, 30, 36, 41].

**Figure 3:**
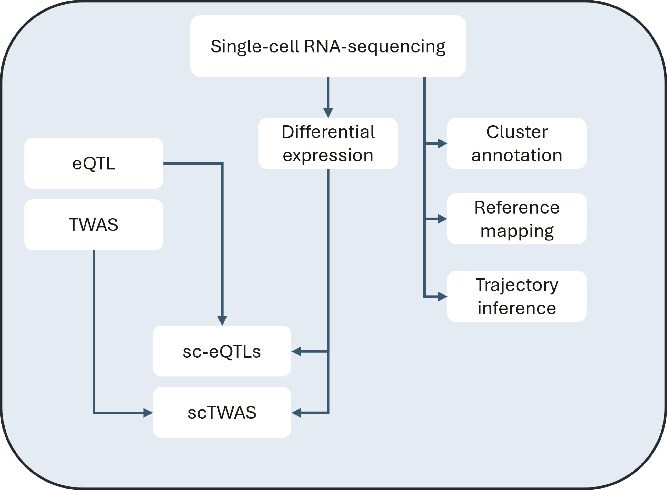
Selected analysis approaches for single-cell RNA data. Single-cell RNA sequencing has multiple additional options for analysis beyond those available for bulk RNA, it also offers benefits in integration with genomics data including extending the usefulness of eQTL and TWAS approaches. The flowchart outlines several common methods of analysis that can be performed with single-cell data and options for integrating single-cell RNA sequencing with genomics in sc-eQTL or scTWAS.

The cell-type-specific nature of scRNA-seq lends itself to integrated network analysis with GWAS and bulk transcriptomics (Figure 3) [38, 108, 110, 111, 121–124]. eQTLs may act in a cell-type-dependent manner; thus, single-cell analyses may reduce the rate of false positives for disease-SNP associations and afford increased statistical power in eQTL detection [103, 110, 111, 121]. For example, GWAS signal enrichment across marker genes for cell clusters can inform the roles of different cell classes in health and production traits for cattle [38, 114, 125]. TWAS is a powerful approach for studying gene-trait associations and may be targeted to specific tissue types through selection of the transcriptome data and gene panels used [85, 104, 105, 107]. Multigroup causal TWAS (M-cTWAS) enables integration of QTL datasets from multiple ‘omics data modalities and contexts such as across different tissues or cell types, showing advantages in terms of higher explained heritability, improved control of false discoveries, and enhanced discovery of risk genes [108]. A natural progression for TWAS is to integrate single-cell data (scTWAS); one approach builds expression models for different classes of cells based on eQTLs identified with single-cell data, while paired genome and single-cell transcriptome data may alternatively be used to directly train prediction models or perform deconvolution of bulk datasets [85, 110, 111]. Advanced methods for scTWAS are particularly useful for identifying cell-type-specific effects and signals; including CONTENT and EXPRESSO, mentioned above [109–111].

Machine learning approaches, including deep learning, have great value in scRNA-seq analysis and have been implemented in many of the methods discussed above [37, 119, 126–129]. There are two main divisions in training: supervised learning where data is labelled, and unsupervised learning where the model identifies patterns in unlabelled data [23, 26, 129–133]. Supervised and semi-supervised approaches generally achieve superior performance on tasks where labels are available, for example in gene regulatory network inference [23, 26]. Deep learning involves training neural networks with multiple layers and may effectively handle large datasets, including automatically extracting and selecting features from complex data [23, 133–137]. Deep unsupervised approaches such as generative adversarial networks (GANs) and autoencoders are very powerful, especially for large datasets where labels may be expensive to generate or unknown; applications include imputation of gene expression values and prediction of disease progression [23, 126, 138–142]. For example, Deng et al. applied an autoencoder-based architecture to develop scScope for cell type prediction from single-cell data [143], while Li et al. combined an autoencoder with a support vector machine for cell type annotation, including discovery of novel cell types, in scRNA-seq data [128]. The trained model should be tested using independent data to assess performance and generalisability [23, 26, 131–133, 144, 145].

Spatio-temporal extensions of scRNA-seq are increasingly important [28, 29, 146–150]. Measuring temporal changes may involve sample collection across multiple timepoints to reveal changes in gene expression and cell composition, while metabolic labelling techniques capture information about RNA transcript kinetics [148, 151, 152]. These approaches are complementary to trajectory inference methods that map cell development across pseudotime, a relative measure of cell progress along inferred differentiation paths [118, 148, 151, 152]. Pseudotime analysis is useful for many applications and complementary to real timepoint analysis, affording increased granularity with many individual cells providing samples of different states along inferred trajectories. RNA velocity measurements can be incorporated to improve the directionality and accuracy of predicted differentiation trajectories [148, 153–155]. However, there are important limitations. Pseudotime methods can struggle to map complex, branching, and non-linear differentiation pathways that occur in immune cells, and network-based methods can be used to integrate gene regulatory network structures generated at different pseudotime points, improving the representation of biological mechanisms and the accuracy of inferred trajectories [24, 148, 154, 156–160]. Challenges include the static nature of the graph representations created, which do not account for the dynamic nature of network connectivity during cell differentiation and state transitions [148, 154–156, 160]. The heterogeneity of cell subpopulations within the tumour microenvironment further complicates trajectory analysis, where environmental factors and cell-cell interactions cause variation in differentiation trajectories [155, 157, 161–166]. Additional challenges include asynchronous patterns such as the cell cycle, feedback mechanisms in immune homeostasis, and rates of apoptosis associated with certain immune cells; these issues should be carefully considered in method selection [155, 157, 161, 167–172].

Spatial transcriptomics approaches capture cellular context within tissues using two main types of methods [173, 174]. Sequencing approaches generally employ arrays of spatially barcoded probes to capture RNA from different regions of the sample [147, 173]. Imaging approaches involve *in situ* hybridisation or sequencing using fluorescent probes specific to individual gene sequences; MERFISH uses multiplexed probe designs with several rounds of hybridisation to identify many genes simultaneously, while STARmap uses multiplexed in situ sequencing with 3D measurement in a hydrogel matrix [175, 176]. Imaging can offer higher resolution than sequencing methods, reaching intracellular levels of detail, and recent developments such as RAEFISH enable whole-genome-level imaging at lower cost [177]. Multiomics approaches may combine measurements for transcriptome, genome, epigenome, and/or protein data [146, 150]. Examples include SNARE-seq [178] for transcriptome and chromatin accessibility, CITE-seq [179] and REAP-seq [180] for transcriptome and protein abundance, TARGET-seq [181] for joint single-cell RNA sequencing and genotyping, scTrio-seq [182] for transcriptome and DNA methylation; and STAMP [183] for sequential multi-modal recording while preserving cell morphology. Co-measurement of different ‘omics datatypes enables more comprehensive profiling of complex biological processes compared to individual techniques in isolation and represents an active area of development [146, 150]. Innovations in spatial ‘omics continue to present bioinformatics challenges, stemming from imaging at subcellular resolution and 3D mapping, as well as cross-platform and multiomics data integration.

Sparsity is critical technical challenge for scRNA-seq data analysis. Zero counts are observed for the majority of gene–cell entries, arising from both genuine transcript absence (“biological zeros”) and technical dropout from incomplete capture or low sequencing depth; the distinction is consequential because the two may require different treatments, and conflating them risks obscuring real biological differences or introducing spurious signal [28, 29, 37, 129, 184, 185]. Recent work has questioned whether classical dropout is a phenomenon distinct from Poisson or negative binomial sampling noise, with droplet-based data better modelled by negative binomial distributions [186]; complementary approaches model zeros within an appropriate count framework and selectively impute likely technical missing data while preserving biological zero values [185]. Imputation methods may be considered in three broad classes with distinct trade-offs: model-free approaches minimise assumptions but can smooth biological variation [28, 184]; model-based statistical approaches encode explicit assumptions about expression distributions; and deep learning methods such as scScope [143], scVI, DCA, and Bubble [142] offer greater flexibility [28, 185, 187, 188].

No single method dominates in scRNA-seq imputation; methods that maximise zero-count recovery may inflate correlations and contribute to false-positive edges in network inference; an important consideration with imputation is the introduction of errors which may increase false-positive rates [188, 189]. scScope is an early deep learning example that imputes missing values as part of its recurrent network architecture that performs cell type clustering and identification as discussed earlier [143]. Bubble similarly applies deep learning with the use of bulk RNA-seq as a reference, restoring cell–cell correlations, with reported low error rates and potential advantages for network inference workflows [142]. Sparsity propagates through downstream analytical steps: in GRN inference, it may bias toward false-negative edges for low-expressed regulators; in trajectory inference, it can produce artefactual branch points; in cell-type assignment, sparse marker genes give noisy cluster boundaries [28, 187–189]. These propagating effects suggest that selection of imputation method should be carefully matched with the downstream analysis task. Model-free approaches are preferable when preserving the correlation structure of the data is the priority, for example in network inference; deep learning methods offer advantages when a well-matched reference dataset is available.

Other challenges for scRNA-seq network inference include technical variability, high dimensionality and batch effects; these issues may be mitigated by optimisation and careful design of experimental methods and analysis workflows [7, 28, 37, 129, 190]. Key tools, resources, and techniques discussed in this and the following section are summarised in Table 1. Different projects may choose to prioritise single-cell or bulk RNA sequencing. The increased expense of single-cell approaches alongside lower throughput and greater data processing complexity are key reasons why bulk sequencing might be chosen. However, the higher resolution of single-cell data is essential for many analyses such as cell heterogeneity, inter-cell dynamics and developmental trajectories [7, 28, 37, 118]. Integration with bulk sequencing may mitigate the higher noise associated with single-cell data and allow greater coverage of RNA transcript and splicing variation [28, 190]. While efforts to improve the sensitivity of single-cell transcriptomics have produced methods such as Smart-seq3 and SCAN-seq2, a combination of single-cell and bulk RNA-seq approaches bring advantages relating to cost and technical complexity [150, 151, 191–193]. Bulk RNA deconvolution involves training models to deconstruct bulk expression data into cell-type-specific values by considering the relative abundance of cell types in single-cell reference data [194, 195]. Application of bulk deconvolution to publicly available datasets offers some of the benefits of single-cell approaches at a lower cost and higher throughput. Bulk RNA may also help inform single-cell data processing, for example in imputation of dropouts with Bubble [28, 142, 184, 185, 187–189].

**Table 1:** Tools, resources and techniques for single-cell transcriptomics and multiomics data generation, processing and downstream analysis.

**Bioinformatics tools and resources**	**Summary**
CellRanger [117]	Processing raw single-cell read data
Seurat [38]	Processing and analysis of single-cell count data including QC, visualisation, clustering, differential expression, cell type identification, and trajectory analysis among other methods.
edgeR [125]	Differential expression analysis for single-cell and bulk RNA count data
Monocle [118]	Processing and analysis of single-cell expression data, including clustering, cell type identification, and pseudotime analysis among other methods.
Scanpy [119]	Processing and analysis of single-cell count data including clustering, visualisation and trajectory inference among other methods.
GF-ICF [120]	Normalises weights and marker genes in single-cell expression data
CONTENT [105]	TWAS analysis with both bulk and single-cell data.
EXPRESSO [109]	sc-TWAS analysis with sc-eQTL summary statistics and integration of 3D genomic data and epigenomic annotation
M-cTWAS [108]	Integrates sets of QTLs across different data modalities and estimates contribution to heritability to identify causal molecular traits and improve causal gene discovery.
scScope [143]	Identification of cell types in single-cell RNA data
Bubble [142]	Performs inference of dropout values in single-cell RNA count data with a paired bulk RNA count matrix
Geneformer [47]	Foundation model trained on collection of single-cell transcriptomes with applications to enhance analysis of smaller domain-specific datasets using transfer learning.
SATURN [48]	Deep learning method for generation of universal cell embeddings and cross-species data integration, encoding biological properties of genes using protein language models. Allows multi-species differential expression analysis.
GenePT [49]	Foundation model for single-cell biology leveraging embeddings from ChatGPT, for use in gene and cell level tasks
Gene Ontology [238]	Curated structured vocabulary developed for describing gene function
KEGG [239]	Database curating descriptions of the function and utilities of different biological systems, including a collection of curated pathways and pathway identifiers to which genes are assigned.
BiNGO [242]	Identifies Gene Ontology (GO) terms that are significantly overrepresented in a gene set
IMMUNETS [19]	A collection of functional gene networks derived from different immune cell types.
scHumanNet [41]	Platform for network analysis of human single-cell RNA data and studying cell-type specific disease associated genes.
scNET [273]	Integrates scRNA-seq with protein-protein interaction networks for identification of cellular pathways and complexes via a GNN method.
NetNC [229]	Active module discovery for gene lists including with paired n=1 samples
GSEA [241]	Data analysis method and software tool to identify enriched pathways from gene expression data
GRNBoost2 [230]	Algorithm for inference of gene regulatory networks with single-cell RNA sequencing data.
**Laboratory techniques**	
Smart-seq3 [192]	Short-read sequencing and analysis method for single cell RNA developed to allow more accurate counting as well as direct Isoform assignment and determination of allelic origin for each molecule
SCAN-seq2 [193]	Single-cell RNA sequencing method to improve accuracy and sensitivity based on long-reads using a third-generation sequencing platform.
MERFISH [175]	Image-based spatial transcriptomics, measuring abundance of RNA molecules using fluorescent probes
STARmap [176]	In-situ sequencing spatial transcriptomics allowing 3-dimensional measurements using a hydrogel matrix
RAEFISH [177]	Image-based spatial transcriptomics using alternative probe design to provide efficient whole transcriptome level analysis
TARGET-Seq [181]	Joint single-cell RNA sequencing and genotyping
SNARE-seq [178]	Joint single-cell RNA sequencing and chromatin accessibility measurement
scTrio-seq [182]	Combined single-cell RNA sequencing and analysis of genomic copy-number variations (CNVs) and the DNA methylome
REAP-seq [180]	Joint single-cell RNA sequencing and measurement of protein abundance
CITE-seq [179]	Joint single-cell RNA sequencing and measurement of protein abundance
STAMP [183]	Sequential recording of multi-modal data including RNA and protein expression while preserving cell morphology

Batch effects can arise from technical differences between platforms, laboratories, and processing conditions. These effects require careful consideration, since technical variation may mask biological signals; on the other hand, batch correction and other processing techniques might lead to data distortion [28, 29, 196–203]. Strategies to guard against batch effects include splitting samples into their components for different processes, ensuring that biological variables are not confounded with batches and taking measurements in tandem such as CITE-seq [179, 202, 204]. Issues with batch effects may also be mitigated by preprocessing with dimensionality reduction and potentially the use of more complex deep learning frameworks tailored to handle sparse, high-dimensional data [143, 205–210]. Batch effects may arise in many different data types, however addressing batch effects in single-cell, including spatial, data is particularly challenging, for example, due to sparsity and the relatively recent emergence of these technologies.

### Immune network biology and precision medicine

The resolution and biological specificity of network models depend directly upon the quality and cell-type resolution of the data considered above; moreover, sparse or bulk-averaged inputs limit the model’s representation scope. These network approaches span co-expression and regulatory inference, context-specific single-cell applications, and deep-learning architectures [5, 19, 20, 24, 41, 126]. Throughout, we consider the advantages and key limitations of current methods.

Mapping information flow and physical interactions between biomolecules across immune cell types and states is central to determining causal relationships between genes or pathways and immune phenotypes such as disease susceptibility. Comparative studies of immune networks across species have revealed both conserved and divergent regulatory mechanisms; while the core transcriptional regulators of immune responses are shared, species-specific differences in cytokine signalling and immune cell differentiation pathways can significantly impact disease susceptibility and therapeutic responses [3, 5, 7, 9–13, 15, 19, 24]. Incorporating interspecies variation into network models may enhance their predictive power and relevance in both human and veterinary medicine. Network biology approaches may be especially helpful in resolving the contributions of genes with limited individual effect sizes, which may be grouped with other contributing genes through higher levels of biological organisation [5, 19, 20, 24, 131, 211]. Indeed, there is extensive crosstalk and linkage between system layers and scales of biology; examples include the effect of concentrations of different metabolites on gene expression, and during infection the competition for resources such as an energy trade-off in producing proteins for milk or for immune cells [2, 212–219]. Efforts to elucidate the complex relationships underpinning cell function have led to development of sophisticated network inference and analysis approaches [20, 24–26, 40, 41, 220].

Gene co-expression methods derive correlations between expression values across conditions to infer biological relationships arising from coregulation, revealing pathway modules and enabling prioritisation of hub genes by graph-theoretic measures such as clustering centrality, betweenness centrality, or node degree [20, 24, 25, 211, 221–227]. Weighted gene co-expression network analysis (WGCNA) is a popular method, though direct application to scRNA-seq is constrained by sparsity [24, 114, 211, 221–223]. Gene co-expression information can inform parameterisation of gene regulatory networks (GRNs), mapping transcription factor–target gene pairings as regulons [20, 25, 26, 228, 229]. GRNBOOST2 [230] and SCENIC+ [231–235] are widely used tools in this context, the latter combines scRNA-seq with TF binding profiles and chromatin accessibility data for GRN inference. SCENIC+ is preferred when chromatin accessibility data are available alongside RNA, while GRNBOOST2 is more broadly applicable to RNA-only datasets and scales more efficiently to large cell numbers.

Network analysis and annotation techniques are available to identify key nodes, clusters, and to explore their functions [20, 25, 211, 222, 225]. Identification of communities or modules has been attempted by a range of methods including Gaussian mixture modelling, random forests, and neural networks [19, 20, 25, 126, 229]. Individual genes may be prioritised according to graph-theoretic characteristics such as clustering centrality, betweenness centrality, or node degree, providing a measure of gene importance in biological pathways, protein complexes, or the influence upon phenotypes [20, 24, 211, 225–227, 236]. Functional annotation analysis informs interpretation and validation of networks, enabling predictions about novel functions in a disease or trait of interest [27, 211, 221, 222, 237]. NetNC supports context-specific active module discovery including analysis of n=1 paired samples, which is useful for precision medicine applications [229]. Genes may be labelled with Gene Ontology terms or KEGG pathway identifiers, enabling evaluation of enrichment across the network or within individual modules; GSEA and the Cytoscape plugin BiNGO are popular approaches for functional annotation of gene lists including network modules [226, 238–243].

**Figure 4:**
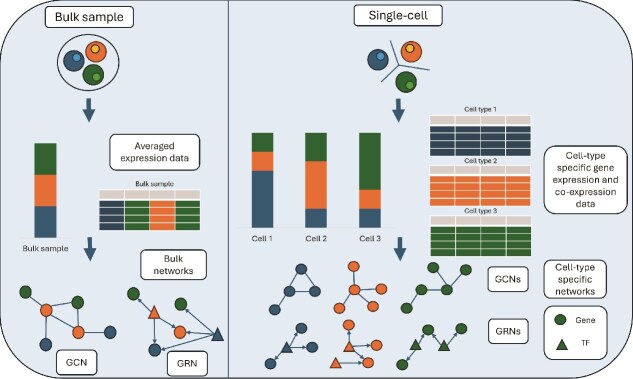
Advantages of single-cell vs bulk data for network modelling. The diagram outlines differences in the application of bulk and single-cell datasets in network biology. Network inference can be conducted with both data types including gene co-expression networks (GCNs) and gene regulatory networks (GRNs). Colours represent the different cell types, their associated expression data and the networks resulting from application of reverse engineering approaches. Bulk sequencing yields average values across different cell types in the sample, leading to network models that may conflate or miss important connections; and may contain structures that do not exist in any specific cell. Expression values derived from single-cell data may produce networks that represent the biology of individual cell types, facilitating clearer understanding of biological mechanisms.

Production of gene networks for specific immune cell types requires a source of context-specific data, such as expression values from individual cell types. An example of this approach produced the IMMUNETS correlation networks for major classes of immune cell types, predicted genes associated with melanoma response to nivolumab and with survival [19]. Single-cell data is highly attractive for this purpose (Figure 4); in neuroblastoma, Verhoeven et al. integrated scRNA-seq data with multiplex immunochemistry to map immune cell landscapes, revealing 27 immune cell subtypes in the neuroblastoma microenvironment including subpopulations of natural killer, B, and T cells [15, 244]. A further example undertook integrative modelling of the developing immune system with scRNA-seq, antigen-receptor sequencing, and spatial transcriptomics of prenatal tissues; mapping more than 100 cell states in space and time, with applications to regenerative medicine and deepening our understanding of congenital disorders [6].

Multiomics resources such as The Cancer Genome Atlas (TCGA) are important foundations for bioinformatics research, including for network biology and precision medicine. Integration of different ‘omics data types alongside clinical outcomes can inform the discovery and validation of candidate biomarkers as well as fundamental biological mechanisms in pan-cancer or cross-species analyses [245–253]. Pan-cancer analysis of TCGA data has characterised the immune landscape across more than 10,000 tumours spanning 33 cancer types, identifying six immune subtypes with distinct macrophage and lymphocyte signatures, neoantigen loads, and prognostic profiles [254]. Computational deconvolution of bulk tumour expression profiles enables estimation of immune cell composition at population scale, as demonstrated across TCGA by correlating immune infiltration patterns with driver mutations, prognosis, and immune subtype characteristics [254]. Machine learning with multiomics for immune network reverse engineering critically requires close alignment of the gold-standard data with the biological context of interest; commonly used datasets including KEGG pathways do not necessarily capture detailed contexts such as the cell type or stage of the cell cycle, and models trained with context-agnostic gold-standard datasets may therefore struggle to identify important biology [255–258]. Cha *et al.* describe scHumanNet, a platform that uses a reference-guided inference approach to construct cell-type-specific networks, which performs well in benchmarking against other single-cell network inference methods in the retrieval of cell-type-specific genes; scHumanNet has been used to generate cell-type-specific gene networks in the HCNetlas database to support discovery of cell-type-specific disease genes and mechanisms [41, 259].

These network resources may be applied to characterise immune cell activity within tumours for precision oncology applications. The activities of infiltrating immune cells within a tumour microenvironment are known to have a key role in determining treatment success and prognosis; for example, immune “hot” tumours have better responses to immunotherapies [19, 260–263]. Many genes have been identified as biomarkers for immune cell activity and immunotherapy response. For example, loss of function in tumour cell B2M or human leukocyte antigen (HLA) class I genes negatively impacts neoantigen presentation to T cells [264–266]; also, mutations in JAK1/2 can inhibit T-cell-induced cell death and abrogate the interferon-gamma-driven upregulation of PD-L1, thus conferring resistance to PD-L1 blockade therapies [261, 263, 267–270]. Network methods have been used in identification of biomarkers associated with immune cells themselves; the IMMUNETS study, noted previously, provides one such example with nivolumab resistance [19].

Regression, correlation, Boolean, and Bayesian approaches are all popular for network modelling from bulk RNA-seq data and have value for single-cell data analysis pipelines [24, 41]. Deep learning methods extend these capabilities by enabling inference and analysis tasks that may be difficult to cast as explicit statistical models. Many studies have also applied deep learning for biological network reconstruction and analysis, both with bulk and single-cell data. Graph neural networks (GNNs) are trained over graph-structured data, learning representations in the form of high-dimensional vectors, and can leverage this graph structure to achieve more accurate and interpretable results across multi-modal data types [126, 134, 271].

GNNs have been applied to cluster gene co-expression network data from Korean cattle, where a graph convolutional neural network (GCN) was deployed to carry out prediction of extracellular gene interactions from spatial single-cell seqFISH+ and MERFISH data [126, 134, 272]. The scNet method uses a GNN architecture to integrate scRNA-seq with protein–protein interaction networks with the intent of providing better characterisation of context-specific gene–gene and cell–cell relationships, as well as to improve pathway analysis and cell clustering [273]. One issue for GNNs in general is a loss of discriminative power as additional layers are added due to over-smoothing, and significant scalability challenges arise as the network size increases [271, 274–276]. Knowledge-primed neural networks (KPNNs) are a deep learning approach which, like GNNs, are applied to graph structures; the trained KPNN represents biological knowledge such as signalling and metabolic pathways, thus enhancing interpretability [127, 277]. A KPNN was constructed to capture biological information flow from receptors to transcription factors and applied to scRNA-seq data for cancer and immune cells, yielding a model of T cell receptor stimulation [127]. While integration of prior knowledge is valuable, existing data sources are typically biased towards known interactions and well-studied genes, therefore limiting exploration of the “dark genome” [278].

Although deep learning and other machine learning approaches are extremely powerful for analysing biological data, large task-relevant datasets are required for effective training. Acquisition of these data can be challenging due to the expense of experimental work, infrastructure, and compute resources needed for large ‘omics projects, as well as limitations in obtaining samples for rare cell types or conditions [47]. Transfer learning can help to overcome a lack of data in machine learning applications, leveraging generally informative and readily available data for pre-training large “foundation models”, followed by fine-tuning with the scarcer, application-specific data [279]. Geneformer, trained on a large corpus of single-cell data for network biology, has been applied to disease modelling with fine-tuning on patient data to discover therapeutic targets [47]. SATURN (Single-cell Analysis via Transfer learning Unsupervised) harnesses single-cell RNA sequencing data and protein embeddings for cross-species analysis of gene functions, including transfer of annotations across large evolutionary distances and multispecies alignment of cell identities [48]. SATURN has been validated primarily on human and mouse data; performance in farm animal species relevant to One Health remains an open question.

Evaluations of SATURN and comparable cross-species methods are predominantly focused upon cell-type annotation tasks; benchmarks for cell-state-specific regulatory transfer, novel-cell-type discovery in non-model species, and species-specific perturbation prediction are less well-developed, and the similarity of embeddings does not always correspond to functional similarity [48, 111]. Alternative approaches use large language models to process gene descriptions and generate single-cell embeddings; the GenePT approach averages literature-based gene embeddings with weightings from expression data and compares well against Geneformer on classification of cell type and gene properties [49]. Geneformer is better suited to tasks requiring expression-level context such as gene network inference; GenePT may be preferred where literature-based gene function descriptions are informative and labelled expression data are limited. Many data sources provide potentially informative priors and may improve performance on certain tasks; however, as with KPNNs, there is a risk of overfitting to prior data or inadequate tuning to the target domain which may limit generalisability and predictive power [280, 281]. These limitations are compounded when models trained predominantly on human or mouse data are applied to other species, where training distributions may be poorly matched to the biological context of interest.

Data quality and availability constrain bioinformatics research, including network inference; existing processing methods developed for bulk data might not work well in single-cell contexts, due to the higher levels of noise and missing values [28, 185, 187–190]. Reducing sparsity with methods such as imputation also has mixed effectiveness due to the potential to introduce bias, including altering the correlation structure of the data [7, 28, 29, 37, 118, 126, 129, 190]. Dataset size is a further consideration, especially in network approaches such as GNNs, which face significant scalability issues known as “neighbour explosion”; mitigation strategies include modifying the model architecture, mini-batch training, and using distributed computation [191, 271, 275, 276]. Overcoming these challenges will be important for the next generation of scRNA-seq network reverse engineering tools, especially when integrating orthogonal multiomics and spatial data at scale.

Analysis that combines different technologies and platforms, even within a single ‘omics type such as transcriptomics, is hindered by inherent differences and biases of the platform or technology used [196, 198, 282]. This issue is readily apparent in the differences between microarray and next-generation sequencing methods, as well as between short and long-read NGS sequencing. Microarray data is affected by several issues including autocorrelations arising from probe locations and ensuring probe target specificity for samples with genetic variation [199, 282]. Sequencing-based methods also face issues, for example large count-based errors for low-expressed genes and factors such as GC content or repeat sequences can impact detection [196, 283, 284]. Furthermore, batch effects may arise for numerous reasons, including environmental conditions and differences between personnel doing the experiment [28, 29, 146, 173, 200, 201]. Detection, mitigation, or ideally prevention of batch effects is crucial to ensure the integrity and usefulness of downstream analysis [28, 29, 146, 173]. Normalisation and other computational approaches are available to remove or reduce batch effects as discussed earlier in relation to single-cell data; care must be taken in method selection because data processing can alter the correlation structure of the data, potentially removing or masking biological signal for network reconstruction [28, 29, 146, 173, 185, 187–189, 203]. Data sparsity and high dimensionality across different ‘omics data types also present challenges for machine learning approaches; models trained on high-dimensional data risk overfitting and so may not generalise well. Approaches to mitigate these issues include preprocessing with dimensionality reduction techniques such as PCA, and potentially the use of learning frameworks tailored for handle sparse, high-dimensional data [143, 206, 207].

Looking forward from network inference methods and associated challenges, we can consider the use of networks not just as descriptive tools, but as foundations for constructing predictive and multi-scale models. Accordingly, the consequences of perturbations such as a new mutation, or a therapeutic intervention, may be simulated *in silico* [42–44, 285–288]. An ultimate aim is to bridge network inference, through dynamic single-cell models, to multi-scale simulations of tissues, organisms, and populations. Model parameters may be formulated in several ways; from discrete thresholds for switching between Boolean activation states, to more complex equations capturing additional physical characteristics such as reaction rates or binding affinities between molecules [42, 43]. Boolean parameterisations offer computational tractability and are suitable for exploratory modelling, while ODE and stochastic formulations are more appropriate when rate constants can be estimated from data and the biological question requires greater quantitative precision or continuous response dynamics [42, 43]. Machine learning methods from regression through to deep learning approaches may be useful for estimating parameter values from ‘omics data and biological networks [42, 44]. Once initial parameters have been established, an executable model allows hypotheses testing and prediction of outcomes, such as a gene’s expression level, a specific disease trait, or the behaviour of a particular gene module [42, 285]. Dynamic models are valuable for studying treatment response or gene perturbation, and for identifying potential disease traits and vulnerabilities, including emergent zoonotic traits in pathogens or synthetic lethal relationships between genes in cancer cells [42, 288].

Agent-based models (ABMs) consist of collections of autonomous agents, each with independent states and parameters that interact to simulate higher-order systems and emergent behaviour [289–291]. Modelling individual system components facilitates capturing heterogeneity, alongside probabilistic rules that reflect the stochastic nature of the underlying biological processes [289–294]. ABMs are well suited to integrating physical interaction rules or spatial data, and modification or addition of individual agents is straightforward with no requirement to alter the rest of the model. ABMs may be applied to a variety of biological scales, from molecules to populations and ecosystems [289–291]. At the intracellular scale, GRNs inferred from scRNA-seq control the internal state of each agent, with edge weights parameterised as Boolean thresholds, ODEs, or stochastic equations. While Boolean parameterisations offer tractability, they may not capture graded responses, ODE formulations capture kinetics but require rate parameters rarely measured directly, and stochastic formulations can better represent single-cell variability [42–44, 285–288]. At the inter-cellular scale, agents may be coupled by contact-dependent signalling, diffusible mediators, and physical constraints, with spatial transcriptomics increasingly used to parameterise co-localisation and ligand–receptor co-expression [177, 183, 272].

The coupling of these models involves important assumptions. For example, when a GRN edge weight inferred from scRNA-seq becomes an intracellular rate or threshold, we assume the regulatory relationship holds under the conditions being simulated; coupling agents via ligand–receptor co-expression assumes that spatial proximity drives signalling. Also, when tissue-level emergent behaviour is used to parameterise population transmission rates, we assume that the *in vitro* or *ex vivo* context of the training data generalises *in vivo*. Few published models have been validated against multi-scale perturbation experiments; indeed, rigorous testing of the above assumptions is challenging [45, 46, 295].

Training predominantly on human or mouse data introduces further limitations; cross-species comparisons of immune networks offer both a test of model generalisability and a route to broader biological insight. Inter-species comparisons have informed on immune system evolution and function, including identification of immune cells [9, 10, 12, 13]. The disease response to certain pathogens can be markedly different across species, as well as pharmacokinetic/pharmacodynamic properties and responses to treatment. These factors are important in drug discovery, due to the widespread use of animal models in disease research [9, 10, 13, 296–301]. Improved modelling of the immune system by considering species-specific differences can help to identify new drug candidates and help avoid missed opportunities due to the limitations of animal models. Immune system differences between hosts and their interactions with pathogens can inform the prediction of diseases with emergent zoonotic potential, through identification of heterogeneity and conserved protection mechanisms between species [9–12, 21, 294, 302–307]. This knowledge is useful in refining multi-scale predictive models, as well as contributing to vaccine development and potentially informing treatments that might be applicable across species [21, 42, 292, 304, 306, 308–318]. Network approaches can help identify key drivers of immune responses and inform rational design of immunotherapies to maximise therapeutic effectiveness [21, 292, 304, 306, 313, 315, 317]. Studies of cross-species immune activation have also revealed host immune molecules that influence pathogen vector immune responses, suggesting additional ways to potentially limit the spread of infectious disease [304].

Species-specific biology is an important challenge in immune research for One Health. Orthology mapping is least reliable in immune gene families that have undergone lineage-specific expansion and functional divergence [319]. Pathogen-driven selection has produced quantitative differences in immune responses both between and within species [320]. Regulatory architecture, including transcription factor binding and enhancer sequences, diverges on shorter evolutionary timescales than protein-coding sequence; for example, demonstrated for liver chromatin across 20 mammalian species [321]. Cell-type composition also differs markedly between species, or example γδ T cells can constitute up to 60–80% of the circulating lymphocyte pool in newborn calves in some breeds compared to a minor population in human peripheral blood [2–4, 8, 322]. Systematic comparison of mouse and human inflammatory responses has also documented substantial divergence under acute stress [323], though this has been contested on the basis of alternative gene-selection criteria. These considerations suggest that cross-species transferability is task-specific: broad cell-type annotations may correlate reasonably well, cell-state-specific regulatory inference is likely less transferable, and aligning rare cell types appears particularly challenging.

These constraints notwithstanding, executable multi-scale models have found application in precision medicine and One Health contexts; virtual cells and digital twins represent the current frontier of this work. Virtual cells may utilise ABMs to predict cellular behaviour, interactions with other cells, and the environment, with applications in simulation of tumours and various other contexts [45, 46, 295, 324–326]. Creating digital twins for patients and different biological systems is a key area of interest in precision medicine, with many potential uses [290, 291, 327–330]. The simulation of drug treatment informed by a patient’s genome or the mutational signature of a particular tumour could allow for more effective tailoring of clinical pathways [44, 45, 324, 331, 332]. Existing applications of digital twins include simulating tumour growth and treatment response, immune activity and interactions between immune cell types, as well as Alzheimer’s disease [333] and immunotherapies [46, 290–292, 295, 324–328, 330]. Modelling the immune system within a digital twin framework could also enable better selective care and breeding of animals, helping to inform optimisation of immune health and productivity [334, 335]. Individual animal genomic and health records are increasingly collected at scale for livestock, representing a tractable starting point for applying agent-based immune modelling to population-level One Health outcomes [80, 335]. Capturing intracellular network dynamics in a multi-scale context allows simulation of emergent system behaviour. For example, ABMs can model the behaviour of cell populations within a tumour, enabling *in silico* exploration of combination therapies that target multiple cell subpopulations, and the investigation of shared mechanisms or neoantigens towards enhanced therapeutic tools [43, 44, 289, 336–341].

**Figure 5:**
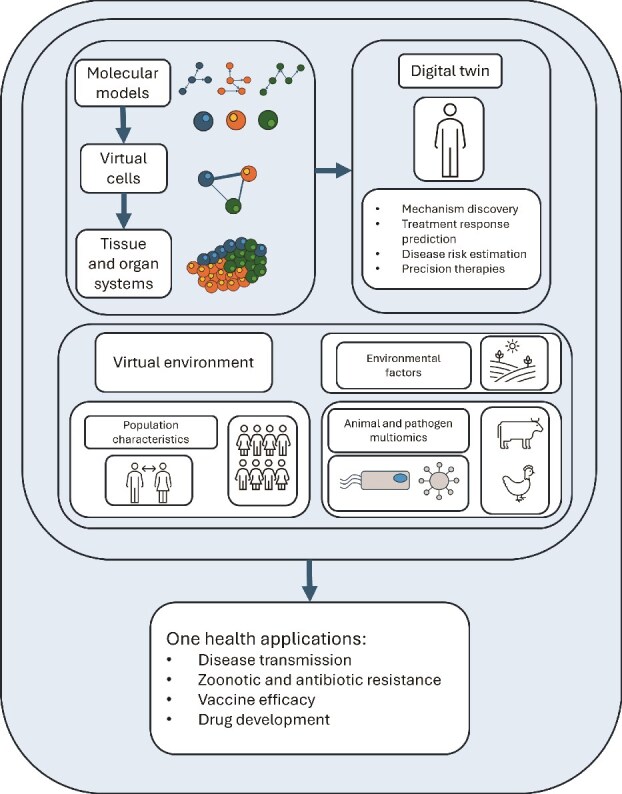
Multiscale and agent-based modelling for one health and precision medicine applications. Biological networks are a useful cornerstone for predictive approaches, including agent-based modelling of multiscale systems from molecules and cells to populations and ecosystems. These models require bioinformatics approaches including machine learning and artificial intelligence applications, to simulate complex systems and emergent behaviour. For example, across ecosystems and considering the influence of the external environment. Virtual cells and digital twins have many potential applications including predicting vulnerability to disease or response to treatment. Digital twins may also predict one health outcomes by incorporating a virtualised context, including environmental factors, population characteristics, and disease vectors.

Digital twins can extend beyond individual organisms or patients to include pathogens, epidemiological metrics, and various other factors to energise One Health approaches (Figure 5) [44, 289, 293, 294, 302, 336, 337]. Applications include modelling population-level immune resilience and tracking disease transmission [44, 45, 293, 294, 302, 303, 328, 336, 342], understanding the spread of zoonotic and antibiotic-resistant disease [293, 294, 302, 303, 342, 343] and studying the effectiveness of vaccination programmes [307, 314, 317, 318, 344, 345]. One Health requires coupling between scales traditionally modelled in isolation: for example, integrating distributions of susceptibility, response kinetics, and pathogen transmission dynamics into population-level simulations [293, 294, 303]. Ecological covariates including climate variables, land-use change, livestock density, and vector distributions can feed back into population susceptibility and transmission parameters [62–64, 68, 293, 342]. As discussed above, these couplings may be formalised with statistical, machine learning or mechanistic approaches.

Accordingly, multiscale modelling brings new challenges, for example arising from the interaction between data sparsity and differences between species. Foundation models such as SATURN are typically evaluated on human or mouse data where coverage is dense. However, performance in farm animal species most relevant to One Health is less well characterised, and sparsity in non-model species may compound issues associated with lineage-specific immune gene evolution and cell-type composition differences [9–13, 48]. Context-agnostic reference data generalises poorly when the biological context of interest is poorly represented [255–258]. Furthermore, errors in sparse or proxy-species input data may propagate through parameterisation and manifest as emergent model behaviours that are difficult to trace or correct [42, 44–46, 285, 295]. As a result, work on an isolated multiscale modelling challenge may have diminishing returns; coordinated development across data quality, evaluation frameworks and model validation may be required to advance the field.

## Conclusion

Modelling context-specific biological states at single-cell resolution is a current challenge in understanding complex processes across scales, including immune responses [5, 6, 19, 21, 38, 40]. Integrated analysis of multiomics data, network approaches and ongoing advances in single-cell technologies all promise improvements in context-specific modelling, to inform upon fundamental biological mechanisms and propose molecular correlates driving clinical outcomes [3, 6, 7, 15, 19, 21, 38, 40]. Indeed, construction and analysis of networks that capture gene function in immune regulation may provide a cipher to unlock connections between genotype and disease phenotypes [3, 5, 19, 21, 124, 211, 224, 225, 227]. For example, enabling deeper interpretation of GWAS and transcriptomics data, which can be used extensively to associate genes with disease trajectories and to illuminate molecular mechanisms [40, 106, 111, 114, 121, 122, 124, 224]. Machine learning, including deep learning, provides a powerful toolkit for data processing, network construction and analysis [23, 26, 126, 127, 134, 143, 144, 272]. This review gives multiple examples of how these methods are deployed with scRNA data to improve the resolution of relationships between genetic variation and neighbouring genes, as well as applications with network inference and machine learning [24, 40, 41, 110, 111, 113, 118, 119, 121, 124, 142, 224]. Key tools, resources and techniques are summarised in Table 1. Network approaches underpin many discoveries, including genes and pathways linked to diseases and phenotypic traits, and they provide a foundation for analysis of immune systems across species. These comparisons have highlighted conserved regulatory modules, such as those governing innate immune responses, as well as species-specific adaptations including differences in cytokine signalling and immune cell plasticity [3, 9–14, 41–44, 77, 92, 102, 113, 114, 121, 131, 285–288, 346]. By integrating these insights, multi-scale predictive models may be refined to better capture immune function and interactions across diverse biological contexts, supporting applications in both human and veterinary medicine. The vast potential areas of application for these methods encompass a variety of different biological contexts from immune resilience to immuno-oncology in humans, cattle and other animals [3, 5–7, 14, 15, 19, 114, 122, 123, 127, 221, 224].

**Key Points**

- Both human and animal populations face significant burdens from disease where the immune system is a key factor.
- The immune system functions through complex interactions between many components and cell types; accordingly, networks are a natural and powerful lens for modelling immune biology in health and disease.
- Whole genome and transcriptome data provide large-scale profiles of the molecular landscape driving cell behaviour; current approaches with single-cell and spatial resolution have further advantages in capturing heterogeneity across cell populations and biological contexts.
- Multi-scale executable models, including agent-based models, virtual cells, and digital twins, bridge molecular network inference and population-level One Health outcomes; with applications in precision medicine, animal health, and zoonotic surveillance.
- Foundation models and transfer learning offer promising routes to propagate biological knowledge to data-poor contexts, including non-model species; however, robust evaluation frameworks for cross-species and cross-context generalisation are still developing.
- Key challenges include data sparsity, context-specific gold-standards, cross-species transferability and multi-scale model validation.

## Data Availability

No new data are reported in this article.
